# In search of universal health coverage – highlighting the accessibility of health care to students with disabilities in Ghana: a qualitative study

**DOI:** 10.1186/s12913-020-05138-0

**Published:** 2020-03-31

**Authors:** Eric Abodey, Irene Vanderpuye, Isaac Mensah, Eric Badu

**Affiliations:** 1 Department of Education and Psychology Studies, University of Cape Coast, Cape Coast, Ghana; 2 Department of Special Education, University of Education, Winneba, Ghana; 3 Department of Health Promotion and Disability Studies; School of Public Health, Kwame Nkrumah University of Science and Technology, Kumasi, Ghana

**Keywords:** Students with disabilities, Accessibility, Health services, Barriers, Health financing, Ghana

## Abstract

**Background:**

Accessibility of health care to students with disabilities is a global concern. This is no less important in Ghana, however, to date, no study has been undertaken regarding access to health care to students with disabilities. This study, therefore, aims to explore the accessibility of health care to students with disabilities, in the quest of achieving universal health coverage in Ghana.

**Methods:**

Qualitative methods, involving in-depth interviews were employed to collect data from 54 participants (29 students with disabilities, 17 health workers and 8 school mothers), selected through purposive sampling. Thematic analysis was used to analyze the data.

**Results:**

The study identified three themes – accessibility, adequacy, and affordability. The study findings highlighted that universal health coverage for students with disabilities has not been achieved due to barriers in accessing health care. The barriers faced by students with disabilities were unfriendly physical environments, structures, equipment, limited support services and poor health insurance policy to finance health care.

**Conclusion:**

The study concludes that the government should prioritize disability-related issues in health policy formulation, implementation and monitoring. The current provisions and requirements in the disability act should be prioritized, enforced and monitored to ensure adequate inclusion of disability issues in health services. Further, the current exemption policy under the National Health Insurance Scheme should be revised to adequately address the needs of people with disabilities.

## Background

The health of an individual is a state of complete physical, mental, and social well-being and not merely the absence of disease or infirmity [1, 2]. Access to health services is recognized as a right to improve the social and economic wellbeing of individuals [2–4]. However, one billion people (constituting 15% of the world’s population) worldwide who have some form of disability [4] experience limited access to social services, including health, education, and economic activities. Worldwide, people with disabilities lag behind the non-disabled population in accessing health services [5–7]. The existing estimates suggest that about 5.8% of persons with disabilities have unmet needs in accessing health services compared with 3.9% of people without disabilities [4]. Several studies have highlighted that people with disabilities in Low and Middle-Income Countries (LMICs) face substantial barriers in accessing health care [8–14]. These barriers are attributed largely to several weaknesses in the health systems as well as individual factors of people with disabilities. The health systems factors contributing to barriers are mostly linked to unfriendly physical, environmental, structural, the unfriendly process of delivering health care, and social barriers (stigmatizing attitudes of health service professionals) [15–20]. The individual factors contributing to barriers are mostly linked to limited awareness about health services, attitudes and beliefs of people with disabilities, limited insurance coverage, and geographical proximity to health service centres [21, 22]. The health systems factors and individual factors jointly contribute to barriers in accessing health services in many LMICs.

Given the above, several efforts have been undertaken to improve the social services (eg. health and education) of people with disabilities. For instance, international declarations such as the Convention of the Rights of People with Disabilities (CRPD) has entrusted countries to improve access to health services for people with disabilities [23]. In particular, the article 25 of the CRPD has tasked governments to ensure that “people with disabilities have the right to the enjoyment of the highest attainable standard of health without discrimination based on disability” [23]. Also, the Salamanca Statement (UNESCO, 1994) together with the CRPD have facilitated policy and advocacy for educating children with disabilities [23, 24]. Further, the recent 2030 agenda (Sustainable Development Targets 4.5 and 4.7) has tasked countries to ensure equal access to all levels of education and vocational training, regardless of disability [25].

Therefore, it has become imperative to understand the health care needs of students with disabilities [26–28]. For instance, listening to students’ experiences, their challenges and needs when accessing health services is an opportunity to examine their priorities and preferences in health service planning. Thus, empirical evidence is needed to explore the accessibility of health services to students with disabilities, particularly in the quest to achieve universal health coverage. Whilst there seems to be increasing evidence on the subject (as part of the broader goal of universal health coverage and inclusive education) [26–28], the empirical studies are limited to developed countries [29], and largely on students with intellectual disabilities, with no study in LMICs like Ghana. The evidence from existing empirical studies conducted in high-income countries are valuable contributions to the literature on access to health services for students with disabilities, yet, they cannot be generalized to LMICs (example, Ghana) due to the differences in the health systems. For instance, developed countries may have adequate resources and social services, compared with developing countries.

In Ghana, the 2010 population census suggests that 737,743 people, representing 3%, of the 30 million population live with some form of disability [30]. The most common disability groups were people with sight (40.1%), hearing (15.0%), speech (13.37%), physical (25.4%), intellectual (15.2%), emotional (18.6%), and other forms of impairment (38.3%) [30]. In accordance with global regulations, several efforts have recently been employed by the Ghanaian government to improve the well-being of people with disabilities. These efforts include the passage of the Persons with Disability Act, 2006 (Act 715) and subsequent ratification of the Act in 2012 [31, 32]. The Act was passed to promote issues such as access to quality health services, adequate medical rehabilitation, employment, rights, education, transportation, housing facilities as well as the participation of persons with disabilities in cultural activities [31, 32]. Per the Act, the National Council on Persons with Disability was established in 2010 to supervise its implementation. As part of the implementation process, people with disabilities were allocated a 3 % share of the District Assembly Common Fund from the Government of Ghana. This fund is said to be used to support access to social services (example, health, and education and income-generating activities) within this population (especially those in the informal sector). However, there are several challenges in the implementation of the fund. For instance, there seems to be a delay in the timely availability of the fund to support people with disabilities. Similarly, the disability common fund appears limited to meet the increasing number of people with disabilities needing such financial assistant. Therefore, people with disabilities are described as the poorest of the poor in the Ghanaian society. In particular, they are ranked low in every economic indicator in the country, particularly, access to education for children with disabilities [21, 33–36].

Consequently, several studies have been undertaken on access to health services for people with disabilities in Ghana [15–18, 20–22, 37, 38]. The studies have documented the barriers and enablers informing access to health services for people with disabilities. However, the studies focus on the Sexual and Reproductive Health (SRH) needs of women with disabilities [22, 39, 40] and deaf patients [15–19]. Also, the evidence focused largely on the general disability population, with relatively no study targeting students. The search of the literature identified few studies that explore social service issues regarding students with disabilities in Ghana but addressed barriers in accessing inclusive education [34, 41–43]. No study has attempted to explore the accessibility of health services for students with disabilities, in the quest of achieving universal health coverage. Students with disabilities may face specific challenges when accessing health services compared with those without disabilities. Specifically, students with disabilities may require inclusive health services that are accessible to meet their needs. They may also require specific support services (for instance, school mothers and preferential treatment) to make the services easily accessible. More so, students with disabilities may require good health and psychological well-being to enable them to achieve academic success, and further transform their social and economic well-being. Therefore, this study aims to contribute to this gap by exploring the accessibility of health care to students with disabilities, in the quest of achieving universal health coverage in Ghana. The study answers the following research questions: 1) what is the accessibility of health care to students with disabilities, 2) what is the affordability of health care to students with disabilities. Further, the study is supported by Pechansky and Thomas’s (1981) theory on access. The theory which initially had four elements/components have recently been expanded into several components, which include availability, accessibility, affordability, adequacy, awareness, acceptability [44–46].

## Methods

### Research participants and sampling

The study employed a qualitative method. The qualitative method helped to explore an in-depth understanding of the experiences of students with disabilities [47–49]. Again, the method enabled the researchers to interact and listen to students with disabilities and capture their experiences. The approach involves an interpretive understanding of the subjective experiences of students with disabilities. The study was conducted between August 2017 and July 2018 in three regions of Ghana (Western Region, Eastern Region and Central Region). The study purposively selected three educational facilities and two health facilities across three regions of Ghana. The educational facilities were purposively selected to capture students with different category of disabilities. The locations of the educational facilities are among the top five regions in Ghana that have the highest population of people with disabilities [30]. The educational facilities recruited for the study were Special Vocational School (in Western Region), School for the Blind (Eastern Region) and School for the Deaf (Central Region). Participants recruited for the study were in-school students with disabilities of 18 years and above (Senior High School level), school mothers as well as health professionals (Medical doctors and Nurses). The school mothers were people who worked in the selected schools to provide care to students. We focused on this group because previous research on vulnerable population in Ghana has concentrated largely on homeless, female head porters (these are young girls who have migrated from northern Ghana to the South because of poverty, marriage pressures and lack of employment) and HIV/AIDs patients [50]. Little is known about students with disabilities, particularly issues about the accessibility to health services. The authors employed several steps to recruit the participants. First, the authors extracted the names of all students with disabilities and school mothers who have visited health facilities in the last year preceding the study from the school records. The researchers purposively selected participants and invited them through a phone call, email and letters. The invitation contained a letter, consent form and participants information sheet explaining the research purpose, how they were selected, the risk and benefits for participating in the study. In cases where any of the participants declined, the researchers replaced them with another person. Overall, only three participants who were approached declined to participate. A total of 54 participants were purposively recruited for the study. Of this, 29 were students with disabilities; 13 students with visual disabilities (from School for the Blind); 11 students with hearing disabilities (from School for the Deaf) and 5 students with intellectual disabilities (from Special Vocational School). Also, 8 school-mothers from Special Vocational and School for the Deaf were purposively selected. The two health facilities selected were located in the Cape Coast Region. The health professionals in the health facilities were selected through purposive sampling. The authors advertised the study through public flyers at the notice board of the health facilities. The public flyers described the inclusion criteria and the research purpose. The researchers purposively selected health professionals who have at least 3 years’ experience providing health services. A total of 17 healthcare professionals (12 nurses and 5 medical doctors) were purposively selected from the two health facilities.

### Data collection methods

In-depth interviews (IDIs) were used to collect data from all the participants. The IDIs allowed the researchers to probe deeply to elicit information which students with disabilities might not have disclosed in the group level discussion [51]. The in-depth interviews were facilitated by an interview guide. The in-depth interviews were conducted by two members of the research team; one read the questions and record (with an audiotape) whilst the other member took notes of all the gestures. For participants with hearing impairments, an open-ended questionnaire was administered with support from a professional sign language interpreter. The sign language interpreter signed the questions to the deaf participants. In addition, the interpreter voiced and recorded the responses from the deaf participants. The in-depth interviews with health professionals and school mothers were held in the English language, which is the primary language of instruction in the educational settings of Ghana. The interviews with students with disabilities were held in Akan (Fante and Twi) (the local dialects of the study region). The researchers had good knowledge (spoken and written) of English and Akan (Fante and Twi) and so had no difficulty with the languages used in the interview. All in-depth interviews were conducted in the selected schools, and hospitals usually at staff common rooms chosen by participants. The interviews were mostly conducted on non-schooling days (eg. Saturdays and Sundays). All interview session lasted for an average of 40 min to 1 h, often at a saturation point (i.e., when no new ideas and issues seemed to arise) [52]. Written informed consent was obtained from all participants prior to their participation in the interviews.

### Research instruments

The study used an open-ended interview guide, developed into a thematic section according to study participants. Questions captured in the interview guide were variables that have been identified by previous literature [15–18, 20–22, 37, 38] and theories [44–46] on accessibility to health care. The interview guide covers sections on the availability, accessibility, adequacy, awareness, affordability, and acceptability (see Additional file 1). The interview guide used several probing questions and clues that prompted the interviewee’s regarding the subject.

### Data analysis

Thematic analysis was used to analyze the data. The thematic analysis is a method for identifying, analyzing, and reporting patterns within the data [53]. The thematic analysis helped to provide vivid descriptions and information regarding students’ experiences when accessing health care [47, 53]. The thematic analysis followed six stages outlined by Braun and Clarke [53], which include familiarization with the data, generating initial codes, searching for themes, reviewing themes, defining and naming themes and producing the report [53]. The authors read all the transcripts many times to familiarize ourselves with the data. The authors met to discuss initial ideas obtain from the reading to develop codes. The coding process describes a formal system to organize the data, develop ideas and thematic description [53]. The authors applied the initial code to a small subset of transcripts. The coding was conducted until a saturation point was reached, where no new ideas emerged from successive reviewing and coding [52, 53]. Upon completing the initial codebook, the researchers reconvened to review and modify the final codebook and also discuss any discrepancies in the coding process.

We employed several steps to integrate the codes and ideas generated from the different participants’ groups. For instance, the generated codes and statements from students with disabilities, school mothers and health professionals were integrated to form a thematic description. The authors combined the perspectives of the participants (students with disabilities, school mothers and health professionals) because they had a common goal towards inclusive health care as well as universal health coverage for students with disabilities.

The thematic descriptions were grouped according to global, organizing, basic themes and codes (see Table 2). The thematic description highlights issues regarding the accessibility of health care to students with disabilities. The organizing themes were supported with verbatim quotes or text from participants.

## Results

### Socio-demographic information

The background information of the participants is presented in Table 1. More than half of the student participants, 53.8 and 66.7%, from the School for the Blind and Special Vocational School were males (see Table 1). The minimum age of the student participants across all the schools was 18 years, but the maximum age varies in all the schools (see Table 1). More than half of health workers, 52.9% were females. The minimum years of working experience was 5 years, whilst the maximum years was 13 years. Also, all the school mother participants were females, with a minimum age of 28 years and the maximum age of 54 years.

**Table 1:** Background information

	Frequency	Percentage (%)
Students with disabilities		
*School for the Blind (n = 13)*		
Gender		
Male	7	53.8
Female	6	46.2
Age		
18–20 years	4	30.8
21–25 years	1	7.7
26–30 years	5	38.5
31 years and above	3	23.1
*School for the Deaf (n = 11)*		
Gender		
Male	5	45.5
Female	6	54.5
Age		
18–20 years	4	36.2
21–25 years	7	63.6
*Special Vocational School (n = 6)*		
Gender		
Male	4	66.7
Female	2	33.3
Age		
18–20 years	2	33.3
21–25 years	3	50.0
26–30 years	1	16.7
*Health workers (n = 17)*		
Health workers participants		
Nurses	12	70.6
Doctors	5	38.5
Gender		
Males	8	47.1
Females	9	52.9
Work experience		
Minimum/Maximum	5/13	
*School mothers (n = 8)*		
Gender		
Females	8	100
Males	–	–
Age		
Minimum/Maximum	28/54	
Education		
Junior High School	2	25.0
Senior High School	3	37.5
Tertiary	3	37.5
Work experience		
Minimum/Maximum	12/22	

### Themes that emerged from the analysis

The study identified three global and seven organizing themes. Global and organizing themes were used to organize the results (see Table 2). The themes were consistent across the student, health professional and school-mother participants. The thematic analysis identified a convergent or similar view from participants regarding the accessibility of health care to students with disabilities. This is because all the participants had a common goal towards the health and well-being of students with disabilities.

**Table 2:** Themes emerging from the analysis

Global themes	Organizing themes	Basic themes	Codes
Theme 1: Accessibility of health care	Physical environment of health facilities	Unfriendly physical environment	• Poor road network • Poor hospital environment
	Physical structure of health facilities	Inaccessible physical structure	• Hospital doors • Hospital building • Overcrowded tables and chairs • Medical tables and chairs • Staircases • Walkways • Unreachable consulting rooms • Malfunctioning lift
	Equipment and logistics	• Inaccessible equipment and logistics	• Directional signs • No tactile or braille
		• Available equipment and logistics	• Limited medical equipment
Theme 2: Adequacy of health care	Support services	• Limited Support services	• No support services • Limited preferential treatment
	Availability of sign language interpreters	• Lack of sign language interpreters	• No sign language interpreters • Communication difficulties • No knowledge of sign language • Miscommunication
		• Limited Sign language support	• School-mothers ability to sign
Theme 3: Affordability of health care	Source of financing health care	• Unreliable financing sources	• Personal funds • Family members • Individual supports • School authority • Social welfare services • National Health Insurance Scheme
	Challenges in financing health care for students with disabilities	• Setbacks in health financing care	• Difficulty in obtaining consultation records • Limited benefit coverage of NHIS • Less expensive drug

### Theme 1: accessibility of health care

#### Physical environment of health facilities

Most participants (students, school mothers and health workers) noted that the physical environments of health facilities were unfriendly to accommodate students with disabilities. The challenges associated with the physical environments were poor road network leading to health facilities, bushy nature of the roads and the immediate hospital environment. Specifically, some participants (students, school mothers and health workers) mentioned that the roads leading to health facilities were untarred, and had open gutters. Some of the participants felt that the poor roads and gutters usually serve as a trap, particularly for students with visual impairments:

*“There are many challenges with the covers on the gutters along the road. The road is not corresponding to the gutters. The gutters are not well covered. Covers connecting the entrance of the hospital are not well cemented, so I have to drag my legs in the pebbles to picture it before I get there”* (Student IDIs, participant 13).

Most participants (students, school mothers and health workers) further expressed that the paths leading to the hospitals were too narrow. This makes movement extremely difficult for students with disabilities, particularly when there is no sighted guide or family caregiver accompanying them to the facility. Besides, school mothers from the selected schools confirmed that the roads leading to health facilities were unfriendly to accommodate students with disabilities.

#### Physical structure of health facilities

The majority of the participants (students and school mothers) had a mixture of feelings about the physical structure of health facilities. Some participants (students, school mothers) noted that the physical structure of health facilities were unfriendly to accommodate students with disabilities. Some participants particularly those with visual impairment felt that they had some difficulty accessing the hospital doors. For instance, some students with visual impairments narrated that the doors were made of sliding glass, but were mostly not open to facilitate their movement:

*“For the blind to access that place, you have to use your hand to locate where the door is, to know if it’s opened or closed. So if it’s opened you just access how big or wide the door is, then you go through”* (Student IDIs, participant 22)*.*

Most participants (students and school mothers) further expressed that there were several obstacles when moving inside the hospital building. Some of the students narrated that the challenges they faced were unfriendly staircase. For instance, the students said there were a lot of staircases in the hospital environment. An unfriendly staircases made it difficult for students with disabilities when walking to and from the consulting rooms and other departments within the health facility:

“When *accessing the Out-Patient Department, I have to climb some stairs, and climbing the stairs is difficult. If you are not careful and the ground is slippery you can easily fall”* (Student IDIs, participant 2).

Some student participants further expressed that there were overcrowded tables and chairs as well as unadjusted medical tables and chairs to meet their needs. Some participants (students with visual impairment) also mentioned that patients without disabilities mostly stood in their walk-ways:

*“One of them is that there are a lot of people in the hospital so some people will be standing in your way not knowing that you are blind and they expect you to see them and swerve them, which I do bump into them. And on the way to the laboratory at the Government Hospital, there are some glasses, going through is difficult at times”* (Student IDIs, participant 16)*.*

Moreover, some participants also expressed that washrooms and toilet facilities in some health facilities were unfriendly to accommodate students with disabilities:

*“and their washrooms are not in a good shape, in that I have to go to a nearby bush to ease myself anytime I visit the hospital”* (Student IDIs, participant 9).

Most health professional participants also confirmed that the health facilities were unfriendly to accommodate students with disabilities, particularly those with visual and physical impairments. Some health professionals noted that students with physical and visual impairments were having difficulty moving around the hospital building:

*“For me honestly, I think the hospital was purposely made not disability friendly. When you go to the Wards, there are some stairs that ideally, it shouldn’t have been there. The washroom has no rails to aid or support the students with disabilities who visit the hospital*” (Health Professional, participant 1).

The experiences of students with disabilities towards the physical structure of health facilities confirm that of the health professionals. For instance, most of the health professionals noted that the consulting rooms of some health facilities were located on a story building, however, the available lift was not always functioning to support students with disabilities. This makes it difficult for students with disabilities, particularly those with visual impairment to access the consulting rooms.

#### Equipment and logistics

The participants shared their experiences regarding the accessibility nature of the existing equipment and logistics in the health facilities. The majority of the participants (students, school mothers) expressed that students with disabilities faced several challenges due to unfriendly equipment and inadequate logistics to support the delivery of accessible health care. Some participants felt that there were no directional signs to support the movement of students with disabilities when accessing health care. Again, the few directional signs were not usually accessible particularly for students with visual impairment. Also, the majority of participants (students, school mothers and health professionals) expressed that there were no tactile or braille to support patients with visual disabilities when accessing health care. This difficulty was also confirmed by one health professional when he stated that” *“There is no tactile system for visually impaired. Those that are in the hospital are not meant for the disabled”* (Health Professional, participant 1).

Although most participants expressed that some health facilities had limited and inaccessible equipment and logistics to support students with disabilities, few health professionals recounted that some equipment and logistics were available to support such patients. For instance, the available equipment was wheelchairs to convey students with disabilities in the hospital. Besides, some health professionals mentioned that students with disabilities particularly those with visual disabilities were given lenses, canes, clutches and stretches to aid their movement in the hospital.

### Theme 2: adequacy of health care

#### Limited support services

This section explored the adequacy of health care to students with disabilities. Here, the participants narrated some of the support services that facilitate access to health care to students with disabilities. In a typical health care practice, students with disabilities (similar to those without disabilities) present to the hospital and give out details of their identity document to the outpatient departments. The outpatient department identifies the medical consultation records of the patients. The nurses then initiate the care and refer the patient to the appropriate medical doctor. The nurses carry the medical consultation records to the consulting rooms on behalf of the patients. Regarding the care, most of the participants expressed that students with disabilities did not receive any special support services. For instance, some participants noted that they had to join the usual queue for hours whenever they visited the outpatients’ services (for instance, getting their medical consultation records):

*“You have to join a queue, and they don’t even consider the fact that you are impaired. There was an instant where I had to stand for about two hours because the seats were full”* (Student IDIs, participant 3)*.*

*“I have familiarized myself with the environment, so I take off those errands by myself by getting my card (consultation records) at the Out-Patient Department (OPD)t. No special support for me”* (Student IDIs, participant 7).

The school-mothers also recounted that there were no special support services provided to students with disabilities when accessing health care. For instance, some school-mothers noted that they experience difficulties when trying to get consultation records (that contain medical history) for students with disabilities at the Out-Patient Department:

*“I put the child on my back and quickly rush to the OPD and get the card (consultation records). At the OPD, the nurses don’t help at all. Unless it is an emergency, but if you go and tell them that O′ Nurse this is a special child, none will help you to process the card (consultation records) or even give you preferential treatment* (School-mother, participant 3).

#### Lack of sign language interpreters

The majority of participants believed that sign language interpreters were valuable in supporting students with hearing disabilities. However, most participants (students, school mothers and health workers) expressed that there were no sign language interpreters in most of the health facilities to support students with hearing disabilities. The participants noted that the lack of sign language interpreters were creating several barriers for students with disabilities. For instance, some participants felt that the absence of sign language interpreters created communication difficulties and subsequently led to miscommunication between patients and health professionals. Some participants expressed that the lack of sign language interpreters sometimes, leads to misinterpretation of the symptoms of illness:

*“I wish they (sign language interpreters) were there because I could have communicated very well since they understand my language and also, I understand theirs. Their absence has caused me a lot, because at times I have to write, which at times I cannot spell the words”* (Student IDIs, participant 19)*.*

*“No interpreters, due to that I have to write and other times I have to go with my parents. Sometimes my writing is not clear to the doctors, and I observed that the doctors are just guessing”* (Student IDIs, participant 23).

The health professionals also expressed that understanding sign language is necessary to facilitate the service delivery, however, they did not know how to sign with students with hearing disabilities. Practically, the lack of knowledge in signing affect how they interpreted students’ diagnosis and illness:

*‘Sign language interpretation is very important, you know we are taking care of a lot of people including those who are deaf and dumb so it will help. Else you can give different diagnosis’* (Health Professional, 6).

Although school-mothers were able to sign and also interpret sign language, the health professionals were deficient in the sign language and its interpretation. This could have a serious consequence on the delivery of health care, particularly to students with hearing disabilities.

### Theme 3: affordability of health care

#### Sources of financing health care

The sources of financing health care is relevant to improve access to health care to students with disabilities. Most participants (students and school mothers) mentioned several sources of financing health care. The students with disabilities financed their health care through personal funds, family members (fathers, mothers, siblings, uncles, aunties and other relatives), individual supports (cooperate entities and individuals), school authority, social workers and National Health Insurance Scheme (NHIS). Most health professional participants recounted that students with disabilities, sometimes, experience difficulty paying for the cost of their health care. This was echoed as follows:

“Some *of the students with disabilities are not able to pay for their bills. Some of them have to beg other patients to foot their bills and sometimes we the nurses have to dip our hands into our pocket”* (Health professional, participant 1).

Although some students with disabilities expressed difficulty in paying for the cost of health care, the health professional participants recounted that there were social welfare services in some health facilities to support students who were unable to pay for their health care expenditure:

*“We have the Social Welfare Department in the hospital. They screen persons who put themselves up not to be able to pay their bills”* (Health professional, participant 1).

#### Challenges in financing health care for students with disabilities.

The participants described some of the challenges associated with the sources of financing health care for students with disabilities. The challenges regarding the sources of financing health care were largely associated with the NHIS. The majority of participants narrated that whilst most students with disabilities were active members of the NHIS, they encountered several difficulties, which include limited benefits and difficulty in obtaining consultation records from the OPD:

*“Acquiring the card (consultation records) is tedious because the process is long. With long queues, where they end up telling you they have a quota they will attend to, leaving the rest”* (Student IDIs, participant 5).

The NHIS appears as a sustainable source of financing health care, yet some mentioned that the scheme had limited benefit coverage and less expensive drugs. Some student participants echoed this as follows:

*“Sometimes you go there and they will tell you the drug they are given you isn’t covered by the NHIS, so you have to go and buy it elsewhere”* (Student IDIs, participant 12)*.*

*“The challenge is that you do not get the best drugs, those you get are just some cheap drugs. You even have to buy some in addition”* (Student IDIs, participant 14)*.*

*“There are a lot of challenges because when you hold the NHIS card (consultation records), they provide you with drugs of less cost and ask you to go buy those that are expensive”* (Student IDIs, participant 4).

The narrations from the student participants were consistent with the challenges expressed by the school mothers regarding the NHIS. For example, some school mothers mentioned that the NHIS benefits were unable to cover some drugs and medications for students, particularly those with intellectual disabilities:

“*Sometimes the medicine to be given is expensive and because you are using the NHIS card (consultation records) they will say that kind of medicine is not available. But, the truth is some will be there. So sometimes when I go I tell them, madam, please if there is some available I am buying it. Because, I am a school-mother and I can’t go and join the queue in a different Drug-store where I would not get”* (School-mother, participant 3).

The analysis implies that the challenges associated with using NHIS could discourage students with disabilities from relying on this source of financing health care. Consequently, students with disabilities may have limited access to health care, which could be associated with their previous experience from the NHIS.

## Discussion

The study aims to explore the accessibility of health care to students with disabilities, in the quest of achieving universal health coverage in Ghana. The study identified three themes, which are consistent with Thomas and Pechansky theory on access to health care. The themes include 1) accessibility of health care, 2) adequacy of health care for students with disabilities and 3) affordability of health care to students with disabilities.

### Accessibility of health care to students with disabilities

Accessibility explains the intersection or fits between the physical location of health services and consumers of services, in terms of physical structure, environment, geographical proximity, travelling time and transportation [44–46]. The health systems that make services easily and readily accessible can increase access to care for students with disabilities. However, the study findings showed that the health care were not accessible to students with disabilities. The students with disabilities faced challenges such as unfriendly physical environments, structures and medical equipment. Consistent with previous studies in LMICs, [22, 37, 39] people with disabilities mostly face several challenges in accessing social services, including health care. The challenges faced by students with disabilities in accessing health care are attributed largely to factors such as health systems weakness, the limited priority of disability issues as well as poor monitoring and evaluation of disability policies. The challenges could influence the quality of health care provided to students with disabilities. In an attempt to achieve universal health coverage, governments in LMICs, including Ghana, should prioritize the health care needs of students with disabilities in policy implementation and monitoring. This can address the needs of students with disabilities and create accessible health systems that are inclusive of the general well-being of people with disabilities.

### Adequacy of health care to students with disabilities

Adequacy (organization) describes the ability to make health care accommodative or well organized to become user-friendly for students with disabilities [44–46]. An adequate inclusion of the needs of people with disabilities into health systems can promote access to health care, irrespective of disability status. The study showed that the health systems had limited provisions to facilitate access to health care for students with disabilities. Importantly, the health system was challenged with inadequate logistics and support services to assist students with disabilities. For instance, assistive support services such as directional signs, tactile or braille, sign language interpreters and preferential services that could facilitate access to care were not available in most of the health facilities. The limited or lack of support services could create a substantial barrier to students with disabilities when accessing health care. Consistent with previous studies, the available health care has limited provisions to support people with disabilities [15, 18, 39]. This challenge is ascribed to limited prioritization of disability issues as well as weak monitoring and enforcement of regulations [15, 18, 39]. For instance, the Persons with Disability Act, 2006 (Act 715) promote access to quality health care, including adequate medical rehabilitation. Also, the Act advocate for the provision of sign language interpreters and other support services to facilitate access to health care. However, close to fourteen years after the passage of the Persons with Disability Act, 2006 (Act 715), the Legislative Instrument that is needed to activate and operationalize aspects of the Act is yet to be passed. There seem to be limited efforts to ensure the enforcement of regulations, partly due to the lack of legislative instrument as well as priority. Given this weakness, there is no specification regarding support services that could promote access to care for students with disabilities [31, 32]. The findings, therefore, recommend that the current policies and strategies, including the provisions and requirements in the persons with disability act 2006 (Act 715), should be revised, prioritized and enforced to ensure an adequate supply of support services. The adequate provisions and prioritization of disability-related issues could help to achieve inclusive health care as well as universal health for students with disabilities.

### Affordability of health care for students with disabilities

The concept of affordability explains the sources of financing health services. Affordability describes the cost of services, ability to pay and existing pre-payment plan [44–46]. The ability to finance health services without out-of-pocket payment can increase access to services for students with disabilities [45]. The study findings suggest that students with disabilities finance the cost of health care through different sources, which included personal funds, family members, individual supports, school support, social workers and NHIS. Despite these, the sources of financing health care were inadequate to support access to care for students with disabilities. For instance, students with disabilities faced several challenges when using NHIS to finance health care. The challenges were attributed to the difficulty in obtaining consultation records at the OPD unit, limited benefit coverage of NHIS, and less expensive drug. This finding confirms previous studies regarding the challenges in financing health care to people with disabilities [21, 22]. As a social protection strategy, the Ghanaian health insurance policy has some exemption criteria for the vulnerable including people with disabilities. However, most people with disabilities are offered this exemption unless they classify themselves as being poor or indigent [21, 38]. People with disabilities are only given some preferential treatment in the process of acquiring and renewing NHIS (for example, skipping queues) but not with the payment of subscription. The challenges in exemption criteria to insurance policy for people with disabilities, together with poor health insurance management practices discourage students with disabilities from using the scheme [22]. The findings recommend that the current exemption policy under the NHIS scheme should be revised to adequately address the needs of students with disabilities. Also, disability advocates (eg. NGOs, policy think-thank) and government agencies are encouraged to promote the inclusion of the needs of students with disabilities in the NHIS policy. Adequate provisions of NHIS services that is responsive to the health financing needs of students with disabilities could help to achieve universal health coverage.

### Strength and limitations

The study has several limitations. The study is limited only to the perspectives of students with disabilities, school mothers and health workers, without the perspectives of stakeholders in government ministries, such as health, education, social protection and regulatory bodies. Also, the interview guide was self-developed by the researchers, without adapting existing validated instruments for measuring access to health services for the vulnerable population. Moreover, the study was limited to only some selected educational and health facilities in three regions of Ghana. Notwithstanding the limitations, the study employed several methodological and interpretive rigour to ensure the trustworthiness of the results. The methodological and interpretive rigour adhere to four principles, including credibility, confirmability, transferability and dependability. The study piloted the interview guide in July 2017 at one educational facility that provide services for students with disabilities. The pilot study interviewed students with disabilities who accessed health services in the last year preceding the study. Again, to increase the confirmability of the study findings, the thematic analysis process were subjected to coding by consensus, member checking, and a series of debriefing sessions [51]. The study findings have been discussed with previous literature on access to health services for people with disabilities.

## Conclusion

The study aims to explore the accessibility of health care to students with disabilities, in the quest of achieving universal health coverage in Ghana. The study findings highlighted that universal health coverage for students with disabilities has not been achieved due to barriers in accessing health care. The barriers faced by students with disabilities were accessibility-related issues, including unfriendly physical environments, structures and equipment. The study findings showed that the available health care has limited support services to facilitate access to health care to students with disabilities. The support services that were lacking in the health facilities were directional signs, tactile or braille, sign language interpreters and preferential treatment services. Further, our study findings demonstrate that the sources of financing health care for students with disabilities were inadequate to facilitate access to care. For instance, students with disabilities faced several challenges when using insurance to finance health care. The health insurance challenges were difficulty in obtaining patients consultation records at the outpatient hospitals, limited benefit coverage of the scheme and less expensive drug.

### Implication for policy and future research

We make several recommendations based on the study findings. The Ghana government should prioritize disability-related issues in health policy formulation, implementation and monitoring. The current provisions and requirements in the Persons with Disability Act, 2006 (Act 715) should be prioritized, enforced and monitored to ensure adequate inclusion of disability issues in social services, including health and education. This could create accessible health care and systems that are inclusive of students with disabilities. Further, the current exemption policy under the NHIS should be revised to adequately address the needs of students with disabilities. The study recommends that future research should aim to explore the preparedness of health workers in providing health care to people with disabilities, including students.

## Supplementary information

**Additional file 1.** Interview Guide.

## Data Availability

The datasets analyzed during the current study is available from the corresponding author on reasonable request.

## Abbreviations

CRPD: Convention of the Rights of People with Disabilities
LMICs: Low and Middle-Income Countries
NHIS: National Health Insurance Scheme
SRH: Sexual and Reproductive Health
IDIs: In-depth Interviews
OPD: Outpatient Department
