# Supplementary material for: In search of universal health coverage – highlighting the accessibility of health care to students with disabilities in Ghana: a qualitative study
Source: BMC Health Serv Res. 2020 Mar 31;20:270. doi: 10.1186/s12913-020-05138-0 (PMC7106671; doi:10.1186/s12913-020-05138-0)
Supplement: Supplementary file 1 — Additional file 1. Interview Guide. [file 12913_2020_5138_MOESM1_ESM.docx]

**Additional file 1: Interview Guide**

**UNIVERSITY OF CAPE COAST**

**COLLEGE OF EDUCATIONAL FOUNDATIONS**

*Thank you very much for your time and interests in this study. This interview schedule is designed to seek views and perceptions concerning access to healthcare services among students with disability. You are being invited to respond to some questions to know your views on the issues. The study is purely for academic purposes and that all the information that you provide will be confidential. For more information and details about the study, Please contact Eric Abodey, (+2330246289287* [onevc2010@yahoo.com or eric.abodey@stu.ucc.edu.gh](mailto:onevc2010@yahoo.com%20or%20%20eric.abodey@stu.ucc.edu.gh)).

| Participant | Question |
| --- | --- |
| Interview guide for Students with disabilities | |
| Demographic information | 1. What is your gender? 2. What is your range? 3. What is your class and programme of study? 4. Which hospital do you visit? 5. Apart from this hospital, is there any other hospital you visit? |
| Interview guide | 1. Tell me about your overall experience when you last visited the health facility to access health care? 2. Tell me about the availability of health care to meet your needs? 3. What are the obstacles/ hindrances/impediments that you encountered when you visited the health facility? 4. Tell me about the inclusivity of the health care to meet your specific health needs? 5. Tell me about the support services available when you access health care in the facilities? 6. Tell me about how you get information about health care? 7. What are the sources of financing health care? 8. What are your experiences with the NHIS as a student with a disability? 9. Tell me about your experiences regarding the attitudes and perception of health provider? |
| Interview guide for Health professionals (medical doctors and nurses) | |
| Demographic information | 1. What is your position in this hospital? 2. How long have you been working here as a nurse/Medical Doctor? |
| Interview guide | 1. Tell me about your experiences when providing health care to students with disabilities? 2. Tell me about the challenges that students with disabilities face when accessing health care? 3. What are the support services that promote access to health care to students with disabilities? |
| Interview guide for School mothers | |
| Demographic information | 1. What is your gender? 2. Please how old are you? 3. What is your level of education? 4. How long have been serving as a house mother? 5. Which hospital do you take the children to when they are sick? |
| Interview guide | 1. Tell me about your experience with access to health care to students with disabilities? 2. What are the specific difficulties people with disabilities face when accessing health care? 3. Tell me about the support services your school provide to facilitate access to health care for students with disabilities? |

***Thank you for your time.***
